# Supplementary material for: Climate Effects on Ergot and Ergot Alkaloids Occurrence in Italian Wheat
Source: Foods. 2024 Jun 17;13(12):1907. doi: 10.3390/foods13121907 (PMC11202928; doi:10.3390/foods13121907)

foods

MDPI

Article

Climate Effects on Ergot and Ergot Alkaloids Occurrence in Italian Wheat

Mariantonietta Peloso [1], Gaetan Minkoumba Sonfack[1], Ilaria Prizio[1], Eleonora Baraldini Molgora[1], Guido Pedretti[2], Giorgio Fedrizzi[1] and Elisabetta Caprai [1], *

[1]Food Chemical Department, IZSLER, via Fiorini 5, Bologna, Italy;
[2]Agronomist Freelance, Como, Italy;

* Correspondence: elisabetta.caprai@izsler.it

1. Supplementary Material

1.1 Tables

Table S1. Temperature, precipitation and relative humidity levels recorded in the province of Lecco between March and July 2023 and average reference values for the area concerned over the last 30 years.

	Period	Temperature (°C)				Precipitation (mm)		Relative Humidity %	
		T-min[1]	T-max[1]	T-rv[2] min	T-rv max	P[1]	P-rv[2]	RH[1]	RH-rv[3]
Lecco province (Lombardy)	March	5.8	17.0	**3.0**	**14.8**	19.8	**63.4**	67.9	**63.5**
	April	8.3	17.2	**6.5**	**18.3**	85.6	**102.7**	65.2	**60.7**
	May	12.4	22.5	**10.7**	**23.4**	179.4	**129.0**	85.1	**62.1**
	June	17.7	28.2	**14.6**	**27.9**	117.4	**101.9**	70.0	**59.4**
	July	20.5	30.0	**16.5**	**30.5**	170.4	**107.7**	76.0	**55.4**

[1] (T) Temperature, (P) cumulative precipitation and (RH) relative humidity % datasets recorded in the area concerned (province of Lecco) between March and July 2023. The data were collected by the ARPA Lombardy sensor at the Montevecchia Cascina Butto (Lecco) meteorological station. [2] (T-rv = temperature reference values; P-rv = Precipitation reference values) The data are compared with average reference values for temperature and cumulative precipitation levels in the area (available data 1991-2023 Vertemate con Minoprio (CO) meteorological station, 30 km from the contaminated field). Average values processed by ERSAF on data from ARPA Lombardy; [3](RH-rv) Relative humidity %, reference value, historical data available for the Bergamo meteorological station (Lombardy) (data ARPA Lombardy 1993-2023).

Table S2. LC conditions for ergot alkaloids analysis

LC conditions	
**LC-MS/MS equipment**	XEVO TQ-Xs Acquity UPLC I Class Plus Waters

**LC Column**	UPLC BEH C18 1.7µm 100 mm x 2.1 mm Waters
**Mobile Phase**	A: 10 mM ammonium carbonate solution (pH 10)<br>B: acetonitrile
**Flow**	0.4 mL/min
**Injection volume**	5µL

Table S3. Gradient elution program for ergot alkaloids analysis

**Time (min)**	**Mobile phase A (%)**	**Mobile Phase B (%)**
0.0	80	20
8.0	30	70
8.5	80	20
11	80	20

Table S4. MS/MS parameters for all analytes (EAs and other mycotoxins)

**MS/MS parameters**	**ERGOT ALKALOIDS**	**OTHER MYCOTOXINS**
Ionization mode	ESI+	ESI+/ESI-
Capillary voltage	0.50 kV	1.00 kV
Source Temperature	150°C	150°
Cone voltage	35 V	40 V
Desolvation Temperature	600°C	600 °C

Table S5. Validation parameters for each EA -ine form at six spiking levels

**Samples**	**Spiking Level µg/kg**	**Mean Conc µg/kg**	**S[1]**	**RSDr[2]**	**Mean Recovery %**
**Ergocornine**	**2**	1.5	0.16	10.79	79.3
	**10**	8.3	0.66	7.90	
	**50**	43.2	4.32	9.99	
	**150**	112.9	5.80	5.14	
	**250**	213.0	17.42	8.18	
	**600**	422.5	36.00	8.52	
**Ergocristine**	**2**	1.4	0.16	11.16	74.7
	**10**	7.4	0.78	10.44	
	**50**	39.5	1.92	4.86	
	**150**	107.2	2.59	2.41	
	**250**	188.9	21.49	11.37	
	**600**	444.1	28.40	6.39	
**Ergocryptine**	**2**	1.4	0.18	12.90	77.4
	**10**	7.7	0.69	8.87	
	**50**	41.4	4.29	10.34	

	**150**	105.1	8.29	7.89	
	**250**	197.9	15.46	7.81	
	**600**	504.7	55.71	11.04	
**Ergosine**	**2**	1.6	0.20	12.33	86.9
	**10**	8.8	0.68	7.77	
	**50**	44.0	3.41	7.74	
	**150**	120.6	6.29	5.22	
	**250**	233.1	21.69	9.30	
	**600**	548.4	62.44	11.38	
**Ergometrine**	**2**	1.6	0.12	7.41	90.7
	**10**	8.5	0.48	5.61	
	**50**	38.1	2.56	6.69	
	**150**	129.8	7.47	5.75	
	**250**	231.5	13.82	5.97	
	**600**	717.8	52.49	7.31	
**Ergotamine**	**2**	1.5	0.25	16.12	76.7
	**10**	7.8	0.82	10.47	
	**50**	39.5	2.70	6.82	
	**150**	107.7	10.31	9.57	
	**250**	202.7	24.59	12.13	
	**600**	439.2	77.67	17.68	

[1] (S) Standard Deviation; [2] (CV%) The Coefficient of Variation of the repeatability was calculated by analyzing blank samples in six replicates at the six fortified levels.

Table S6. Validation parameters for each EA -inine form at six spiking levels

Samples	Spiking Level μg/kg	Mean conc μg/kg	S[1]	RSDr[2]	Mean Recovery %
**Ergocorninine**	**2**	1.6	0.19	11.50	88.9
	**10**	8.9	0.64	7.11	
	**50**	47.6	6.41	13.46	
	**150**	121.3	8.70	7.17	
	**250**	230.5	30.00	13.01	
	**600**	549.7	71.81	13.06	
**Ergocristinine**	**2**	1.5	0.18	11.69	80.7
	**10**	8.2	0.83	10.02	
	**50**	43.9	3.78	8.60	
	**150**	108.3	7.33	6.77	
	**250**	199.5	19.41	9.73	
	**600**	515.2	55.73	10.82	
**Ergocryptinine**	**2**	1.7	0.14	7.56	79.8
	**10**	8.0	0.61	7.53	
	**50**	41.1	4.27	10.38	
	**150**	105.0	4.91	4.67	

	**250**	195.9	16.91	8.63	
	**600**	466.6	50.25	10.77	
**Ergosinine**	**2**	1.7	0.13	7.62	91.2
	**10**	9.0	0.47	5.21	
	**50**	46.4	3.96	8.53	
	**150**	126.6	8.71	6.88	
	**250**	237.2	22.02	9.28	
	**600**	594.6	39.68	6.67	
**Ergometrinine**	**2**	1.6	0.12	7.01	91.1
	**10**	8.7	0.52	6.01	
	**50**	41.4	2.92	7.04	
	**150**	129.6	10.10	7.79	
	**250**	231.5	14.91	6.52	
	**600**	717.8	52.08	7.55	
**Ergotaminine**	**2**	1.6	0.14	8.54	87.2
	**10**	8.9	0.74	8.33	
	**50**	45.1	3.56	7.89	
	**150**	116.9	7.63	6.52	
	**250**	214.2	14.81	6.91	
	**600**	580.0	40.39	6.96	

[1] **(S) Standard Deviation;** [2] **(CV%) The** Coefficient of Variation of the repeatability was calculated by analyzing blank samples in six replicates at the six fortified levels.

Table S7. Validation parameters for EAs sum

Samples	Spiking Level µg/kg	Mean conc µg/kg	S[1]	RSDr[2]	Mean Recovery %
**EAs sum**	**24**	19	1.77	9.2	83.7
	**120**	101	7.35	7.3	
	**600**	512	37.28	7.3	
	**1,800**	1,392	72.85	5.2	
	**3,000**	2,574	204.20	7.9	
	**7,200**	6,473	388.05	6.0	

[1] (S) Standard Deviation; [2] (CV%) The Coefficient of Variation of the repeatability was calculated by analyzing blank samples in six replicates at the six fortified levels.

Table S8. LC-MS/MS parameters for other mycotoxins

Mycotoxins	ESI	Precursor Ion (m/z)	Product Ion (m/z) Q/q[1]
Aflatoxins (B1, B2, G1 and G2)	+	B1 313.1	241.0/269.0
		B2 314.9	259.0/243.0
		G1 328.9	243.0/199.8
		G2 330.9	189.0/217.0
Ochratoxin A	+	403.9	239.2/357.8

Deoxynivalenol	+	297.4	249.4/231.1
Zearalenone	-	317.30	130.9/160.7
Fumonisins (B1 e B2)	+	B1 722.4	352.3/334.2
		B2 706.4	336.3/318.9
T2 toxin	+	484.2	185.1/215.1
HT2 toxin	+	442.3	263.1/215.1

[1]Q=qualifier, q=quantifier

Table S9. LC conditions for mycotoxins analysis

LC conditions	
**LC-MS/MS equipment**	XEVO TQ-Xs Acquity UPLC I Class Plus Waters
**LC Column**	Acquity UPLC BEH C8 1.7µm 100 mm x 2.1 mm Waters
**Mobile Phase**	A: water/acetonitrile 0.1% formic acid (95/5)<br>B: acetonitrile 0.1% formic acid
**Flow**	0.4 mL/min
**Injection volume**	5µL

Table S10. Gradient elution program (mycotoxins)

Time (min)	Mobile phase A (%)	Mobile Phase B (%)
0.0	70	30
0.1	70	30
3.0	20	80
3.5	70	30
4.0	70	30

Table S11. Total content of 12 ergot alkaloids in sclerotia samples and corresponding wet weight.

Samples number[1]	Wet Weight (g)	T-EAs[2] (µg/kg)
**S1**	0.24	1,158,760
**S2**	0.32	698,640
**S3**	0.20	819,530
**S4**	0.20	110,090
**S5**	0.36	639,100
**S6**	0.18	1,421,970
**S7**	0.08	147,050
**S8**	0.07	698,960
**S9**	0.07	337,720
**S10**	0.08	702,140
**S11**	0.06	4,951,190
**S12**	0.02	1,019,080
**S13**	0.03	1,208,110
**S14**	0.03	1,218,340

**S15**	0.01	2,590
**WE1**	1.00	2,300
**WE2**	0.52	70
**WE3**	0.23	120
**WE4**	0.47	50
**WE5**	0.49	60
**WE6**	0.14	30
**WE7**	0.36	10
**WE8**	0.76	20
**WE9**	0.63	1,450
**WE10**	0.36	33,110
**WE11**	0.66	40
**WK1**	10.00	1,150

[1] S= sclerotia; WE= wheat ear; WK= wheat kernels; [2] (T-EAs) Total content of ergot alkaloids

Table S12. The concentrations of ergot alkaloids (-ine form) in analyzed samples

**Samples number**[1]	**Ergocornine μg/kg**	**Ergocristine μg/kg**	**Ergocryptine μg/kg**	**Ergosine μg/kg**	**Ergometrine μg/kg**	**Ergotamine μg/kg**
**S1**	10,872.0	187,479.0	136,652.0	259,960.0	102,561.0	56,559.0
**S2**	< LOQ[2]	210,342.0	1,859.0	97,553.0	167.0	133,249.0
**S3**	< LOQ	216,586.0	614.0	30,381.0	77,210.0	123,333.0
**S4**	1,207.0	64.0	23,074.0	25,161.0	2,965.0	92.0
**S5**	75,324.0	24,035.0	41,668.0	154,380.0	59,333.0	9,224.0
**S6**	< LOQ	440,337.1	3,317.1	337,221.6	124,045.8	220,254.2
**S7**	< LOQ	56,821.4	398.5	19,582.4	69.4	28,221.4
**S8**	142,094.4	13,137.1	88,984.2	174,590.5	38,327.0	9,275.7
**S9**	2,155.5	72,791.4	2,060.0	92,845.9	36,023.5	13,778.5
**S10**	203,251.3	984.2	107,525.7	143,216.2	29,852.9	1,244.2

**S11**	1,001,533.3	190.0	623,237.1	705,720.2	251,147.0	1,331.4
**S12**	45,901.3	124.2	331,617.1	16,454.0	111,205.8	34.2
**S13**	335,620.8	65.7	150,771.4	38,762.1	225,564.1	312.8
**S14**	121,872.0	39.0	175,879.0	86,869.0	355,865.0	422.0
**S15**	180.5	88.5	1,194.2	294.5	288.2	85.7
**WE1**	118.0	82.0	56.0	432.0	674.0	110.0
**WE2**	5.0	< LOQ	< LOQ	4.0	40.0	< LOQ
**WE3**	9.0	< LOQ	4.0	10.0	55.0	< LOQ
**WE4**	5.0	< LOQ	< LOQ	6.0	22.0	< LOQ
**WE5**	< LOQ	< LOQ	5.0	4.0	33.0	< LOQ
**WE6**	< LOQ	< LOQ	< LOQ	< LOQ	26.0	< LOQ
**WE7**	< LOQ	< LOQ	< LOQ	< LOQ	8.0	< LOQ
**WE8**	< LOQ	< LOQ	< LOQ	< LOQ	12.0	< LOQ
**WE9**	3.00	< LOQ	18.0	947.0	30.0	< LOQ
**WE10**	1,037.0	1,930.0	13,230.0	2,240.0	1,680.0	466.0
**WE11**	3.0	< LOQ	10.0	5.0	14.0	< LOQ
**WK1**	3.0	350.0	24.0	181.0	148.0	68.0

[1] S= sclerotia; WE= wheat ear; WK= wheat kernels; [2]LOQ= 2 μg/kg

Table S13. The concentrations of ergot alkaloids (-inine form) in analyzed samples

**Samples number**[1]	**Ergocorninine µg/kg**	**Ergocristinine µg/kg**	**Ergocryptinine µg/kg**	**Ergosinine µg/kg**	**Ergometrinine µg/kg**	**Ergotaminine µg/kg**
**S1**	10,559.0	95,926.0	83,574.0	122,834.0	70,986.0	20,802.0
**S2**	< LOQ[2]	138,875.0	1,364.0	50,141.0	130.0	64,958.0
**S3**	< LOQ	216,664.0	641.0	17,975.0	62,254.0	73,870.0
**S4**	3,013.0	67.0	32,031.0	17,584.0	4,791.0	41.0
**S5**	86,187.0	18,347.0	35,447.0	87,265.0	44,131.0	3,758.0
**S6**	844.0	146,938.5	892.9	68,664.9	36,152.0	43,304.2
**S7**	< LOQ	24,827.7	192.9	7,414.9	20.0	9,504.2
**S8**	92,710.7	4,330.8	40,957.1	67,154.1	24,540.0	2,861.1
**S9**	2,028.0	40,096.9	1,501.4	43,527.0	25,221.3	5,688.9
**S10**	107,953.3	426.2	41,721.4	52,867.6	12,734.7	361.1
**S11**	114,2236.0	126.2	630,792.9	436,247.3	157,321.3	1,305.6
**S12**	65,685.3	133.8	303,358.6	57,189.2	87,372.0	< LOQ
**S13**	211,729.3	23.1	74,328.6	34,589.2	136,224.0	122.2
**S14**	113,326.0	13.0	148,029.0	41,836.0	174,002.0	188.0
**S15**	108.0	36.9	85.7	97.3	129.3	< LOQ
**WE1**	118.0	75.0	48.0	238.0	298.0	53.0
**WE2**	3.0	< LOQ	< LOQ	< LOQ	21.0	< LOQ
**WE3**	8.0	< LOQ	< LOQ	5.0	33.0	< LOQ

**WE4**	< LOQ	< LOQ	< LOQ	3.0	10.0	< LOQ
**WE5**	< LOQ	< LOQ	< LOQ	< LOQ	17.0	< LOQ
**WE6**	< LOQ	< LOQ	< LOQ	< LOQ	8.0	< LOQ
**WE7**	< LOQ	< LOQ	< LOQ	< LOQ	4.0	< LOQ
**WE8**	< LOQ	< LOQ	< LOQ	< LOQ	7.0	< LOQ
**WE9**	< LOQ	< LOQ	15.0	421.0	18.0	< LOQ
**WE10**	846.0	1,488.0	7,732.0	1,127.0	1,136.0	196.0
**WE11**	< LOQ	< LOQ	< LOQ	< LOQ	8.0	< LOQ
**WK1**	3.0	181.0	13.0	83.0	75.0	25.0

[1] S= sclerotia; WE= wheat ear; WK= wheat kernels; [2]LOQ= 2 μg/kg

Table S14. Total content of other mycotoxins in wheat kernels sample

Mycotoxins	LOQ (μg/kg)	Mycotoxins Concentration (μg/kg)
Aflatoxins (B1, B2, G1 and G2)	0.5	< LOQ
Ochratoxin A	1	< LOQ
Deoxynivalenol	50	2251[1] ± 562
Zearalenone	10	< LOQ
Fumonisins (B1 and B2)	25	< LOQ
T2 toxin	5	< LOQ
HT2 toxin	5	< LOQ

[1]The value exceeds the maximum level of 1750 μg/kg for unprocessed durum wheat kernels, for human consumption, set by EU Regulation 2023/915

1.2 Figures

Figure S1. Chromatograms of ergocristine, ergocryptine and corresponding epimers at 3 ng/mL

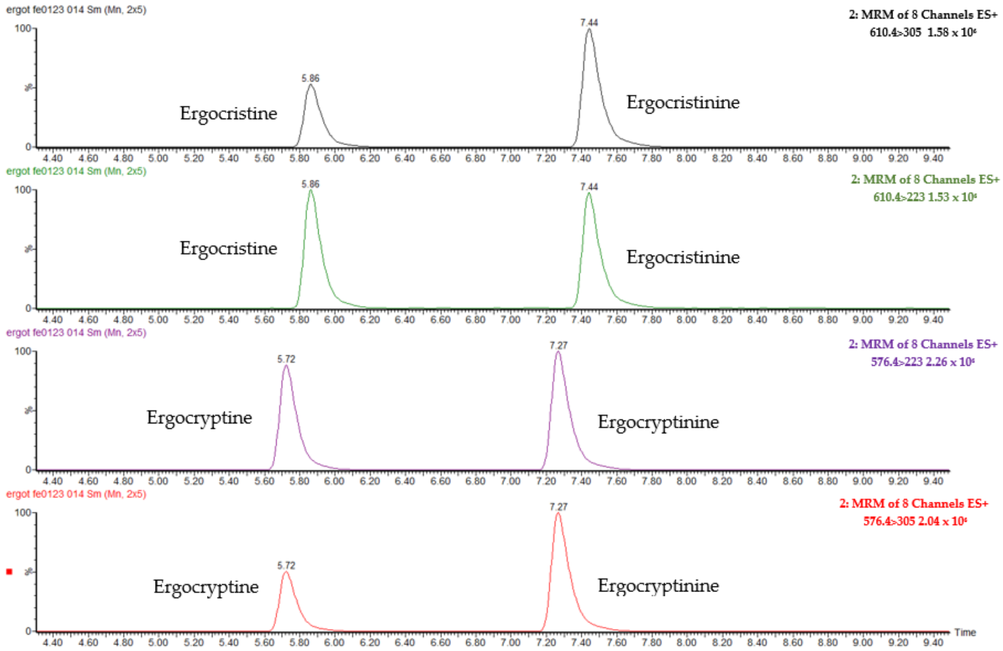

Figure S2. Chromatograms of ergosine, ergocornine and corresponding epimers at 3 ng/mL

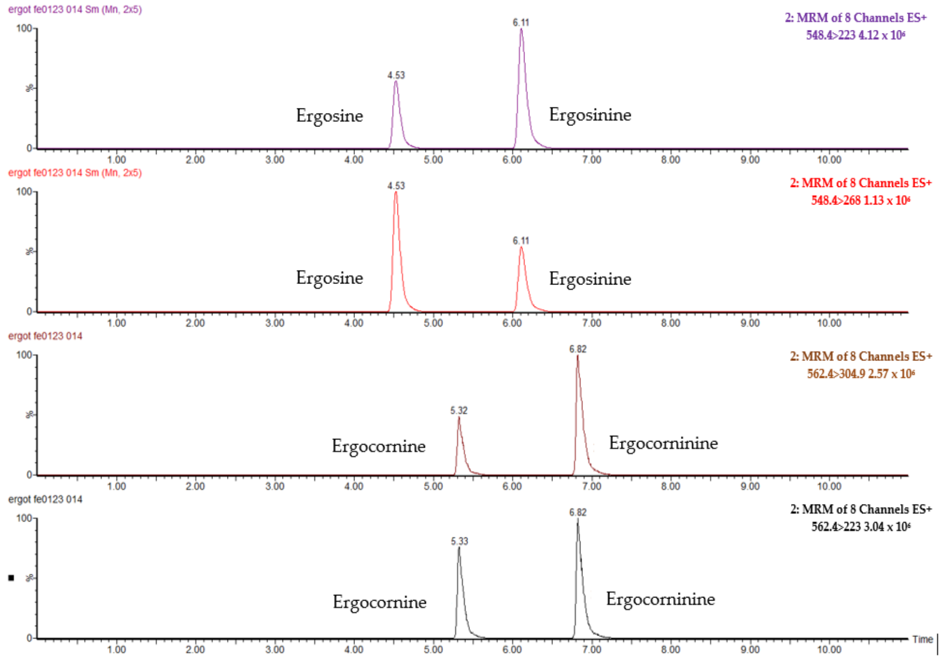

Figure S3. Chromatograms of ergotamine, ergometrine and corresponding epimers at 3 ng/mL

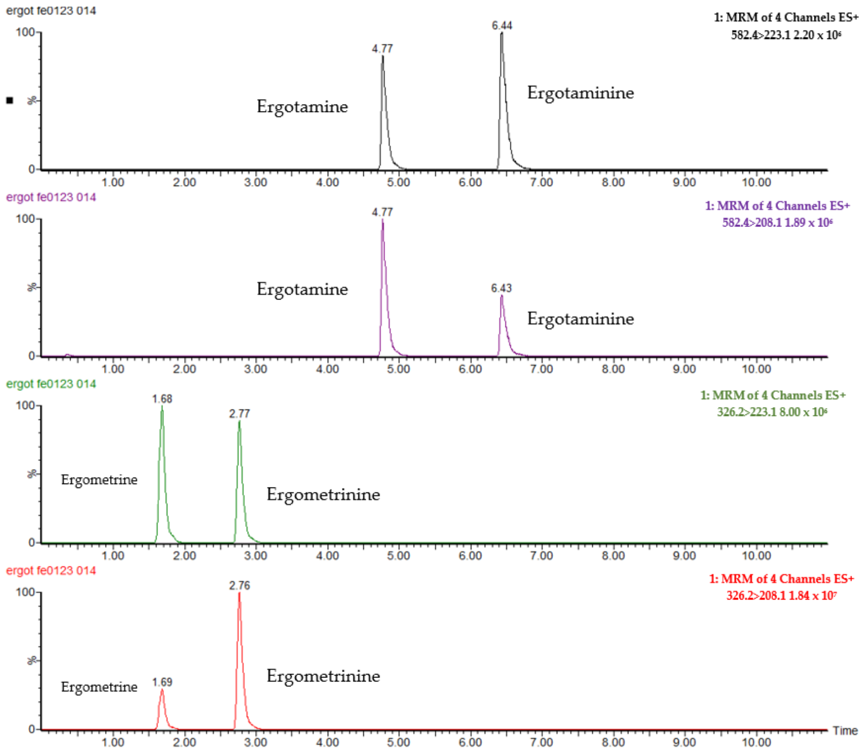

Supplement: Supplementary file 1 [file foods-13-01907-s001.zip › foods-3018843-supplementary.pdf]
